# Circular RNAs at the crossroads of acute pancreatitis: bridging molecular mechanisms to diagnostic and therapeutic innovations

**DOI:** 10.3389/fgene.2025.1650363

**Published:** 2025-11-24

**Authors:** Jing Chen, XiaoLing Chen, Fei He

**Affiliations:** 1 College of Stomatology, Xi’an Jiaotong University, Xi’an, China; 2 Key Laboratory of Shaanxi Province for Craniofacial Precision Medicine Research, College of Stomatology, Xi’an Jiaotong University, Xi’an, China; 3 Department of Oral and Maxillofacial Surgery, College of Stomatology, Xi’an Jiaotong University, Xi’an, China; 4 Department of Surgery, Institute of Integrated Traditional Chinese and Western Medicine, Frontiers Science Center for Disease-related Molecular Network, West China Hospital, Sichuan University, Chengdu, China; 5 Department of Neurology, School of medicine, Sichuan provincial People’s Hospital, University of Electronic Science and Technology of China, Chengdu, China

**Keywords:** CircRNAs, acute pancreatitis, biomarker, therapeutic tool, chronic pancreaititis

## Abstract

Acute pancreatitis (AP) is a severe inflammatory condition that frequently leads to systemic inflammatory response syndrome and multiple organ failure. Despite the increasing occurrence, targeted therapies remain unavailable. CircRNAs have recently been implicated in AP pathogenesis, regulating biological processes such as apoptosis, pyroptosis, and inflammation through mechanisms including miRNA sponging, protein scaffolding, and translation. Their disease-specific roles make them promising candidates for clinical translation. This review highlights the potential of circRNAs as novel diagnostic tools and therapeutic targets in AP, aiming to guide future advancement of innovative biomarkers and therapeutic strategies.

## Graphical Abstract

**Figure:**
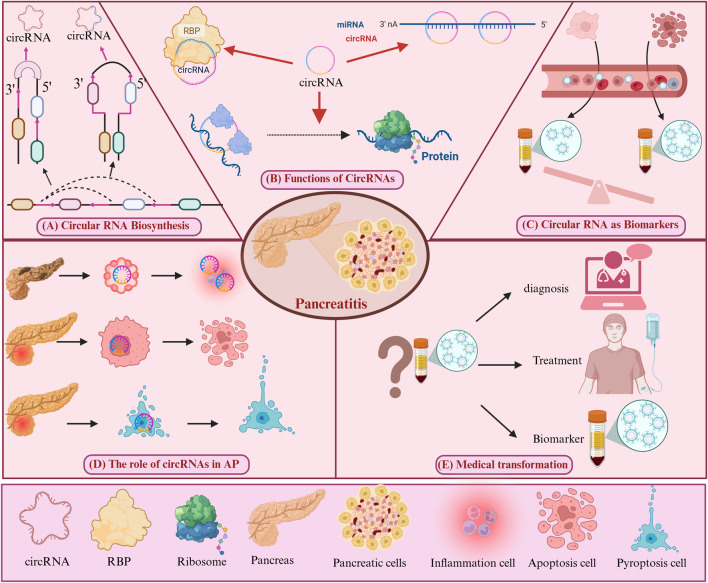
circRNA’s roles in acute pancreatitis pathogenesis and medical prospects.

## 1 Introduction

With a 1%–5% case fatality rate in extreme cases, acute pancreatitis (AP)-a condition characterized by inflammation of the pancreas-manifests as severe abdominal pain and can progress to multiple organ failure and pancreatic necrosis (Petrov and Yadav, 2019). The primary pathological mechanism involves premature activation of pancreatic digestive enzymes, causing inflammation and autodigestion (Boxhoorn et al., 2020), Additional contributors include aberrant calcium ion levels (Huang et al., 2017; Sutton, 2020), oxidative stress, and inflammatory cell infiltration (Sendler et al., 2013; Sendler et al., 2020). Patients may experience persistent pain, recurrent episodes, and long-term functional impairment, which considerably reduce quality of life and increase socioeconomic burden. AP exhibits a broad spectrum of severity, ranging from mild manifestations to life-threatening complications.

Circular RNAs (circRNAs) are a class of single-stranded, covalently closed RNAs made up of exons, introns, or a combination of both (Zhang et al., 2016). They are produced through back-splicing (Zhang et al., 2016) or via lariat-derived RNA intermediates (Jeck and Sharpless, 2014). While the vast majority of circRNAs are considered non-protein-coding and function as miRNA sponges, a subset of circRNAs has been found to possess coding potential and can be translated into functional peptides or proteins (Du et al., 2017a). These molecules hold significant clinical potential and play essential roles in cell growth and proliferation.

While numerous reviews have summarized the roles of circRNAs across different diseases, this article focuses specifically on their implications in AP, offering a systematic integration of circRNA mechanisms—such as miRNA sponging and translational regulation—within the AP context. Furthermore, we emphasize the translational potential, discussing recent advances and preclinical evidence that support circRNAs as both biomarkers and therapeutic targets for AP. For instance, circRNAs contribute significantly to AP pathogenesis, by c modulating crucial signaling pathways including inflammation, apoptosis, and pyroptosis. Moreover, circRNAs have emerged as promising diagnostic biomarkers and therapeutic targets for the early detection of AP (Liu C. et al., 2020), owing to their exceptional stability conferred by the covalently closed circular structure (which confers resistance to RNase R degradation) and their highly tissue-specific expression patterns. As a case in point, a study by Liu et al. (2020) demonstrated the utility of differentially expressed circRNAs in the plasma of AP patients for early diagnosis.

Despite the accumulating literature in this area, a dedicated review that synthesizes the current knowledge of circRNAs specifically in AP—particularly regarding their mechanisms and clinical applicability—is lacking. Therefore, this review aims to particularly regarding their mechanisms summarizing the functional roles and regulatory mechanisms of circRNAs in AP and evaluating their potential for diagnosis and targeted therapy, ultimately providing a foundational resource for future research and clinical translation.

### 1.1 Literature search and selection

This narrative review synthesizes current evidence on the roles and mechanistic insights of circular RNAs (circRNAs) in acute pancreatitis (AP). Literature searches were performed in electronic databases, including PubMed and Web of Science, using key terms such as “circular RNAs,” “circRNAs,” “acute pancreatitis,” and related synonyms. Study selection adhered to the following criteria:

Inclusion criteria: (1) original research focusing on the function, mechanism, or clinical relevance of circRNAs in AP; (2) studies conducted in human subjects, animal models, or cell lines; and (3) articles published in peer-reviewed journals.

Exclusion criteria: (1) conference abstracts, reviews, editorials, or dissertations; (2) articles whose full text was inaccessible or contained non-extractable data; and (3) studies irrelevant to AP.

Owing to the emerging nature of circRNAs research in AP, the search yielded a circumscribed body of core literature that met these criteria, all of which has been incorporated into this synthesis.

## 2 Circular RNA

### 2.1 Circular RNA biosynthesis

Two processes are involved in the synthesis of circRNAs: the lariat-driven pathway (Jeck and Sharpless, 2014), and direct back-splicing (Zhang et al., 2016; Chen, 2020). Direct back-splicing is facilitated by complementary sequences within lengthy flanking introns (Liang and Wilusz, 2014) or through the dimerization of RNA-binding proteins (RBPs) (Kristensen et al., 2019). These complementary sequences, known as reverse complementary matches (RCMs), which can be either repeated or non-repeated elements (Ivanov et al., 2015), are essential for circular RNAs formation. Inverted repeating Alu elements are particularly common RCMs. Furthermore, RBPs such as Quaking (QKI) (Conn et al., 2015), Muscleblind (MBL) (Liang et al., 2017), FUS-binding protein (Errichelli et al., 2017), and NF90/NF110 proteins (Li et al., 2017) are known to attach to the introns on both sides of the exons to generate circRNAs, hence increasing circRNAs creation in RBP dimerization. In contrast to canonical back-splicing, circRNAs can be generated from lariat structure, especially when introns lack inverted repeat sequences like Alu elements (Jeck and Sharpless, 2014). In such cases, the lariat may contain exons. The lariat can produce an exonic circular RNA if it is not broken down but instead processed further (Barrett et al., 2015).

**FIGURE 1:**
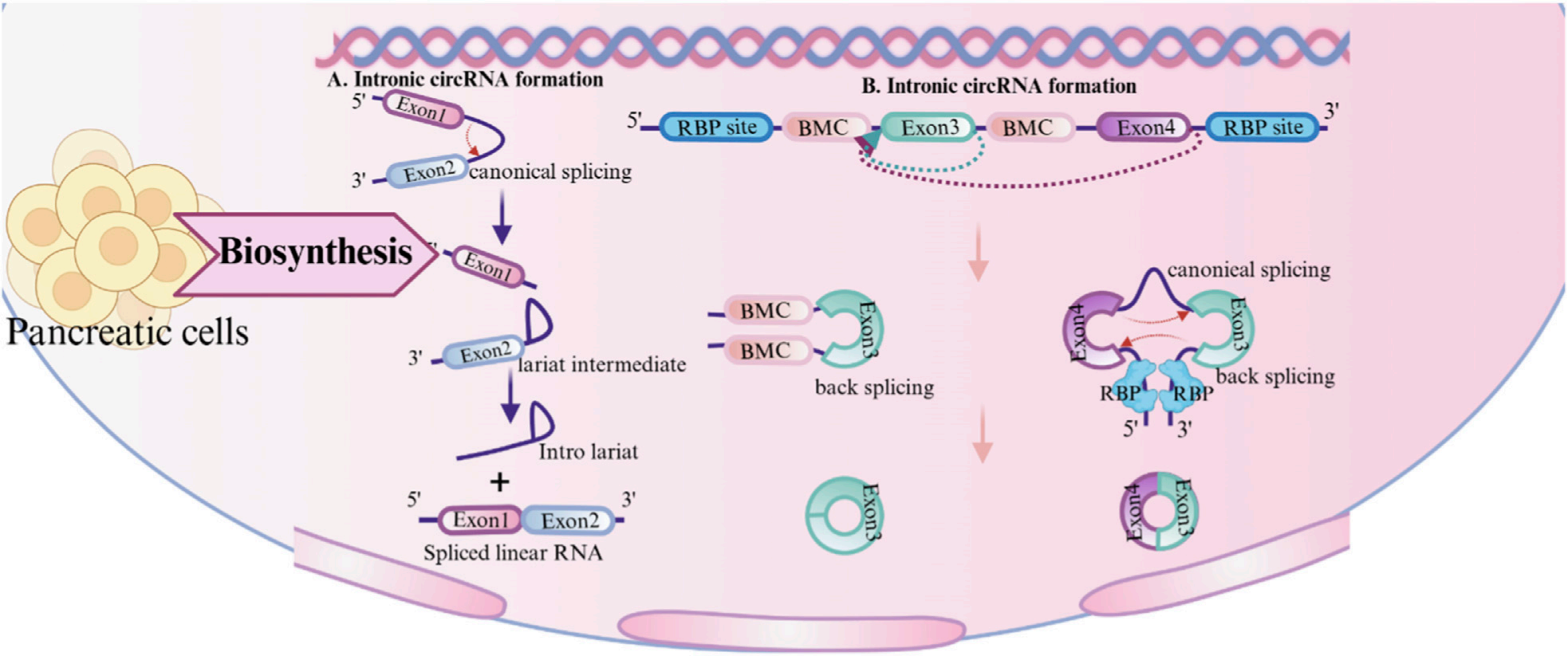
Synthesis of circular RNA (circRNA). circRNA: circular RNA; RBP: RNA-binding protein; RCM: Reverse complementary matches.

Based on genomic origin, circRNAs are primarily classified into four types: exonic (EcircRNAs) (Jeck et al., 2013), intronic (IcircRNAs) (Zhang et al., 2013), exon-intron (EIciRNAs) (Salzman et al., 2013), and intergenic circRNAs (PMC, 2025). While the majority of circRNAs are generated through pre-mRNA back-splicing, a minor fraction originates from lariat introns via linear splicing. (Figure 1). In addition to this classification, circRNAs can be categorized by the genomes of subcellular organelles. For instance, mitochondrial genome-encoded circRNAs (mecciRNAs) have been identified and implicated in regulating mitochondrial function and cardiac pathology (Liu et al., 2025). Furthermore, the mitochondrial circRNA SCAR (steatohepatitis-associated circRNA ATP5B regulator) plays crucial cellular roles in various tissues (Wu et al., 2020; Zhao et al., 2020; Costa et al., 2021; Wu et al., 2023).

### 2.2 Regulation of circRNAs synthesis

Beyond the core mechanisms of back-splicing and RBP modulation, circRNAs biogenesis is further shaped by structural and sequence features. Longer exons exhibit increased susceptibility to circularization due to the higher availability of splice sites, thereby enhancing circRNAs formation efficiency (Liang and Wilusz, 2014). Suboptimal base pairing in intronic repeats—where GU mismatches replace canonical Watson-Crick pairs—can still promote the biogenesis of circRNAs by enabling partial RNA duplex formation, thus facilitating splice site recognition (Liang and Wilusz, 2014). The presence of polyadenylation signals competes with back-splicing machinery, as these elements may redirect pre-mRNA processing toward linear splicing pathways, thereby suppressing circRNAs generation (Liang and Wilusz, 2014). Notably, adenosine-to-inosine (A-to-I) RNA editing mediated by Adenosine Deaminase Acting on RNA 1 (ADAR1) disrupts double-stranded RNA (dsRNA) structures (e.g., reverse complementary matches between intronic sequences), thereby preventing RNA-binding protein (RBP) recruitment or proper alignment of splice sites, which ultimately inhibits circRNAs formation (Ivanov et al., 2015) (Figure 1).

### 2.3 CircRNAs degradation

Circular RNA degradation can be divided into four categories according to the auxiliary factors needed: structure-mediated, m^6^A-mediated, RNase L-mediated, and miRNA-mediated (Liu et al., 2019). Due to their covalently closed circular structure, circRNAs are highly stable, and their degradation primarily relies on endonucleases that catalyze internal cleavage. For example, the circRNA CDR1as/ciRS-7 can recruit miR-671-loaded Ago2 to CDR1as/ciRS-7, causing Ago2 to cleave the molecule endonucleolytically, which in turn causes exonucleolytic RNA degradation (Hansen et al., 2011). Additionally, m^6^A-modified circRNAs can recruit the adaptor protein HRSP12 through the m^6^A mark;, HRSP12 then bridges endoribonuclease RNase P/MRP and the m^6^A reader protein YTHDF2, thereby initiating circRNAs degradation (Park et al., 2019). In contrast, the mechanism underlying RNase L-mediated circRNAs degradation remains poorly understood, though Liu et al. have proposed that it may be related to circRNAs tending to form a 16–26-base pair (bp) imperfect RNA duplex (Liu et al., 2019). Finally, structure-dependent decay involves the UPF1(Up-frameshift 1) RNA helicase and G3BP1(GTPase-activating protein SH3 domain-binding protein 1) stress granule assembly factor, which facilitate the degradation of circRNAs (Fischer et al., 2020).

## 3 Functions of CircRNAs

### 3.1 miRNA sponge

**FIGURE 2:**
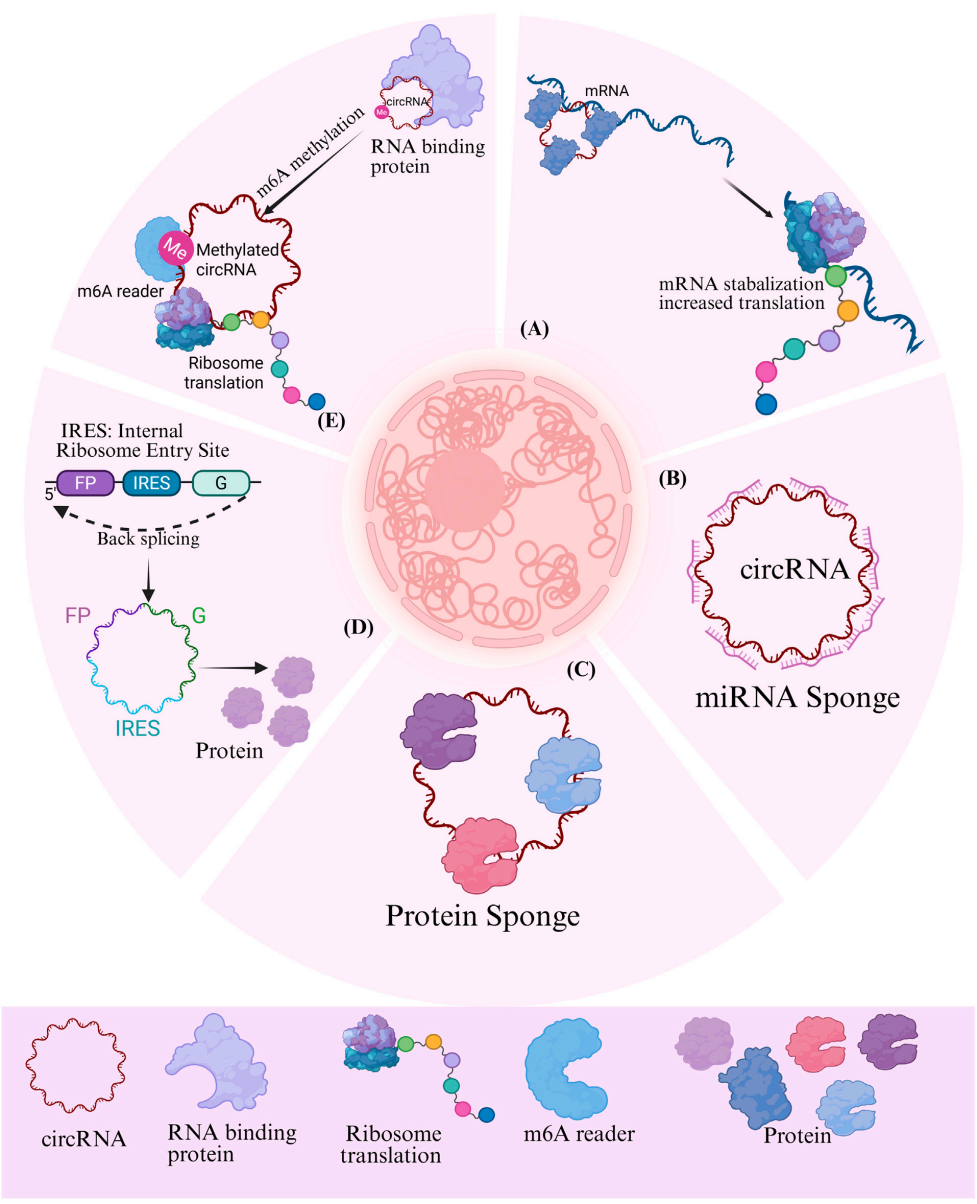
Potential role of circRNAs in the development and progression of pancreatitis. m6A: methylated N6 adenosine; IRES: internal ribosomal entry site; IL F2: Interleukin Enhancer-Binding Factor 2/Nuclear Factor 45; IL F3: Interleukin Enhancer-Binding Factor 3/Nuclear Factor 90; MBL: Muscleblind-like protein.

CircRNAs mostly operate through interactions with other molecules, with the most widely studied mechanism being miRNA sponges (Panda, 2018; Thomson and Dinger, 2016). For instance, ciRS-7, a prominent circRNAs, possesses over 70 conserved binding sites of miR-7, enabling it to regulate the expression of its target genes by interaction with miR-7 (Hansen et al., 2013; Memczak et al., 2013). The effect of ciRS-7 on miR-7 (either inhibition or protection) may be contingent upon the specific cellular milieu (Kleaveland et al., 2018). Moreover, additional circRNAs, including circHIPK3 (Zheng et al., 2016), circPVT1 (Verduci et al., 2017), and circBIRC6 (Yu et al., 2017), have been validated to possess analogous miRNA sponge capabilities, therefore elucidating the diverse role of circRNAs in gene regulation (Figure 2).

### 3.2 Protein modulator

The function interactions between circRNAs and proteins can be categorized into several key mechanistic themes. (Zhou et al., 2020). First, circRNAs can serve as scaffolds to facilitate protein-protein interactions, thereby promoting post-translational modification or trans-activation of the bound proteins (Du et al., 2017b; H et al., 2019). Second, they can act as decoys by binding to proteins and and inhibiting their interactions with other biomolecules, such as DNA (Xia et al., 2018; Chen et al., 2016),RNA (Barbagallo et al., 2018; Barbagallo et al., 2019), and other proteins (Xueting et al., 2019). Third, circRNAs participate in epigenetic regulation by recruiting transcription factors, nucleosome remodeling complexes (Chen et al., 2018; Liu B. et al., 2020), or modifying enzymes to specific genomic loci (Jie et al., 2020). Additionally, circRNAs can form complexes with proteins and mRNA to either increase translation by stabilizing mRNA (Guarnerio et al., 2019) or repressing it (Chen et al., 2020). Finally, the subcellular localization of circRNAs regulating proteins, such as nuclear circRNAs associated with proteins (Yang Q. et al., 2017), cytoplasm circRNAs facilitating nuclear protein input (Yang Z-G. et al., 2017) or altering protein distribution through nuclear export mechanism (Wang S. et al., 2019).

### 3.3 Transcriptional regulation

CircRNAs influence gene transcription through several distinct mechanisms. First, they can bind to transcription factors and act as co-activators to directly modulate transcriptional activity (Li Z. et al., 2015; Guarnerio et al., 2020). Second, by competing with linear mRNAs for splice sites, circRNAs can promote exon skipping, thereby altering splicing patterns and gene expression outcomes (Liang et al., 2017; Ashwal-Fluss et al., 2014; Kelly et al., 2015; Conn et al., 2017). Third, interactions with specific RNA-binding proteins can allow circRNAs to alter the regulatory functions of these proteins (Ashwal-Fluss et al., 2014). Finally, circRNAs can indirectly influence gene expression by sequestering miRNAs (Xia et al., 2018).

### 3.4 Template for translation

The absence of a 5′cap and 3′polyadenylate tail necessitates unique processes for the translation of circRNAs. At present, two recognized forms of circRNAs translation exist: m6A-dependent translation and IRES-mediated translation. Research indicates that m6A-modified circRNAs can initiate translation through the collaborative function of m6A reading proteins (e.g., YTHDF3) and translation initiation factors (e.g., eIF4G2) (Yang Y. et al., 2017; Di Timoteo et al., 2020). IRES translations are either endogenous or synthetically engineered circRNAs that incorporate IRES. Ribosomes can be directly recruited for translation (Macejak and Sarnow, 1991; Chen and Sarnow, 1995; Zhang et al., 2020). It is noteworthy that these two mechanisms can be synergistic: m6A alteration can improve IRES activity, hence dramatically enhancing the translation efficiency of circRNAs (Di Timoteo et al., 2020; Legnini et al., 2017) (Figure 2).

## 4 The role of circRNAs methylation modification in AP

N6-methyladenosine (m6A) modification is prevalent in eukaryotic mRNA, but its mechanism of action in circRNAs remains ambiguous. Research indicates that m6A modification in circRNAs is governed by the same “writer” (e.g., METTL3), “eraser” (e.g., FTO), and “reader” (e.g., YTHDF2) as mRNA (Zhang et al., 2020; Shulman and Stern-Ginossar, 2020), and exhibits specificity for cell type (Zhou et al., 2017). This modification critically influences the fate of circRNAs by governing two opposing outcomes: cap-independent translation (Yang Y. et al., 2017) and RNA decay (Park et al., 2019) (Ries et al., 2019). On one hand, m6A modification can promote the translation of circRNAs. By recruiting initiation factors and the ribosomal complex through YTHDF family readers, m6A drives cap-independent translation, enabling a subset of circRNAs to produce functional peptides that contribute to disease pathogenesis (Yang Y. et al., 2017). On the other hand, m6A-modified circRNAs can also undergo accelerate the degradation. For instance, RNase P/MRP complexes or reader proteins such as YTHDF2 can recognize m6A-modified circRNAs and facilitate their decay. Additionally, certain m6A-modified circRNAs are susceptible to cleavage by RNase P/MRP complexes (Park et al., 2019), indicating a dynamic and context-dependent regulatory layer (Zhang et al., 2020; Shulman and Stern-Ginossar, 2020).

A study in a severe acute pancreatitis (SAP) mouse model identified 57 circRNAs with aberrant m6A modification, which were implicated in pathological processes such as autophagy (Wu et al., 2021). For example, the constructed m6A-circRNA-miRNA network indicated that miR-24-3p and miR-26a may modulate disease progression through their interaction with m6A-circRNAs (Shrinivas et al., 2019) (Wu et al., 2021). Furthermore, the demethylase ALKBH5 (alkylation repair homolog protein 5) was found to be upregulated in this model, suggesting that active m6A demethylation contributes to SAP progression (Wu et al., 2021).

These findings highlight the m6A-circRNA axis as a promising therapeutic frontier. Testable hypotheses can now be formulated: for example, does ALKBH5 gain-of-function in pancreatic acinar cells exacerbate AP by reshaping the m6A landscape of key circRNAs? Conversely, could inhibiting ALKBH5 or therapeutically targeting m6A-modified circRNAs present a viable strategy to ameliorate SAP? Future investigations to validate these mechanisms in human samples and through *in vivo* functional studies will be crucial for translating these insights into novel diagnostics and therapies approaches.

## 5 Circular RNA as biomarkers

Exosomes, which are frequently found in blood and other body fluids (Ma et al., 2019), serve as reliable extracellular transporters for proteins, nucleic acids, and other bioactive molecules (van Niel et al., 2018). CircRNAs are abundantly present in extracellular vesicles (Li Y. et al., 2015; Wang Y. et al., 2019), and their expression profiles can differentiate between healthy and diseased states, highlighting their potential as diagnostic biomarkers (Li Y. et al., 2015).

CircRNAs can be used as predictive biomarkers as well as diagnostic biomarkers. For instance, postoperative atrial fibrillation can be predicted based on the presence of particular circRNAs in the patient’s plasma. Furthermore, the presence of specific circRNAs in the plasma following the initiation of the disease can also predict organ dysfunction (Vausort et al., 2016; Plasma Circular RNAs, 2016). In AP, recent clinical evidence confirms circRNAs as promising diagnostic markers. A study profiling blood circRNAs in AP patients identified six differentially expressed circRNAs (e.g., hsa_circRNA_101015, hsa_circRNA_101211, hsa_circRNA_103470), three of which significantly increased with disease severity and showed high diagnostic accuracy via receiver operating characteristic (ROC) analysis (Liu C. et al., 2020). This establishes circRNAs as AP-specific biomarkers.

However, while circRNAs hold promise as diagnostic biomarkers for AP, they should be evaluated against established diagnostic markers such as amylase, lipase, C-reactive protein (CRP), and clinical scoring systems like BISAP (Bedside Index for Severity in Acute Pancreatitis) and Ranson scores. Amylase and lipase have long been the gold standard for diagnosing AP, though their diagnostic accuracy can be limited by sensitivity and specificity, especially in mild cases or delayed presentations (Guda et al., 2018). CRP is widely used to assess the inflammation level and severity, but it is a non-specific acute-phase reactant that can be elevated in a wide array of other inflammatory and infectious conditions, limiting its specificity for AP (Rm et al., 2016). The BISAP and Ranson scores are valuable for predicting the severity and mortality of AP, but they rely on a combination of clinical and laboratory parameters that require 24–48 h to complete and can be complex for rapid bedside application (Mounzer et al., 2012).

Comparatively, circRNAs may offer more specificity and sensitivity due to their unique expression patterns in AP. Recent studies have demonstrated that circRNAs are not only upregulated in AP but also correlate strongly with disease severity, offering an early and non-invasive diagnostic tool (Yang et al., 2020). Furthermore, circRNAs can be detected in exosomes, which are stable and easy to isolate from blood or plasma samples, presenting a significant advantage over traditional markers that may require serial measurements and lack organ specificity (Li Y. et al., 2015; Wang Y. et al., 2019). This suggests that integrating circRNAs into diagnostic panels could complement or even surpass the current biomarkers in terms of both accuracy and ease of use, particularly when combined with other markers in multi-biomarker approaches (Liu C. et al., 2020).

To benchmark circRNAs against existing biomarkers, further large-scale clinical studies are required. These studies should compare the diagnostic accuracy, sensitivity, specificity, and prognostic value of circRNAs with conventional biomarkers like amylase, lipase, CRP, and the BISAP/Ranson scores. Additionally, evaluating the potential of circRNAs in combination with these existing markers could improve the overall diagnostic and prognostic capabilities for AP.

## 6 The role of circRNAs in AP

**FIGURE 3:**
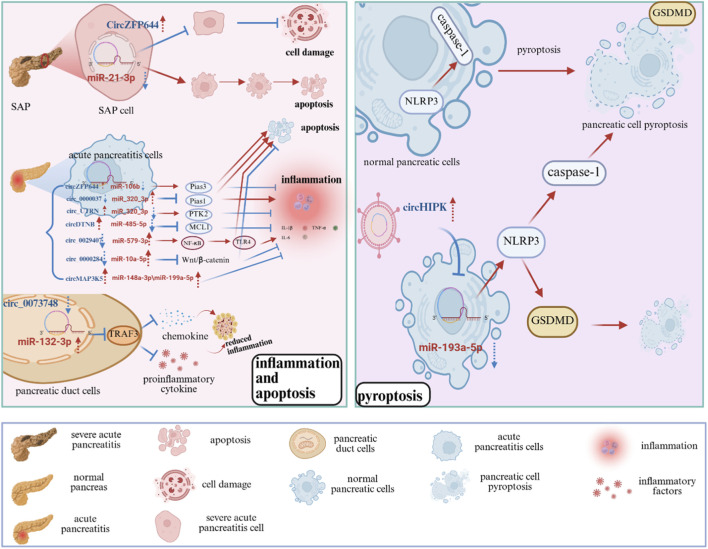
Potential role of circRNAs in the development and progression of pancreatitis. The role of circRNAs in AP, involving key processes such as inflammation, apoptosis, and pyroptosis, and highlighting their potential as diagnostic biomarkers and therapeutic targets. SAP: Severe Acute Pancreatitis; Pias3: Protein Inhibitor of Activated STAT 3; MCL1: Myeloid Cell Leukemia 1; NLRP3: NOD-, LRR- and pyrin domain-containing protein 3; GSDMD: Gasdermin D; TRAF3: TNF Receptor-Associated Factor 3; PTK2: Protein Tyrosine Kinase 2; TNF-α: Tumor Necrosis Factor-alpha; IL-1β: Interleukin-1 beta; IL-6: Interleukin-6; NF-κB: Nuclear factor kappa-light-chain-enhancer of activated B cells; TLR4: Toll-Like Receptor four.

CircRNAs participate in the occurrence and progression of AP by regulating inflammatory responses, cell death, epigenetic modifications, and other critical mechanisms, with their aberrant expression intimately linked to disease progression. This article provides a comprehensive analysis of the association between circRNAs and pancreatitis, examining inflammation, apoptosis, and pyroptosis in detail (Figure 3; Table 1).

**TABLE 1:** The expression and targets of AP associated circRNA’s.

Phenotype	CircRNA	Disease stage	Type of sample (human/Animals/Cells)	Study size	Study type	Evidence level	Expression	Targets	The roles in pancreatitis	References
Inflammation	RNA hsa_circ_0073748	AP (mild/early)	Human, HPDE6-C7 cell	29 patients +19 controls; cell experiment	Clin + Cae (*in vitro*)	Level 1 (Strong)	Increased	miR-132-3p/TRAF3/NF-κB Axis	Alleviation	Ren et al. (2022)
	Circ_UTRN	AP (early/acute phase)	AR42J Cell	Only cell experiments	Cae (*in vitro*)	Level 3 (Basic)	Decreased	miR-320-3p/PTK2 Axis	Alleviation	Sun et al. (2022)
	Mmu_circ_0000037	AP (early/acute phase)	MPC-83 Cell	Only cell experiments	Cae (*in vitro*)	Level 3 (Basic)	Decreased	miR-92a-3p/Pias1 Axis	Alleviation	Chen et al. (2023b)
	Circ_0029407	AP (early/acute phase)	Human, HPECs Cell	19patients +21rols; cell experiment	Clin + Cae (*in vitro*)	Level 1 (Strong)	Increased	miR-579-3p/TLR4/NF-κB Axis	Aggravatin	Lu et al. (2024)
	CircMAP3K5	AP (covering mild, moderate, and severe cases)	Human, MPC-83 Cell	10 mild AP + 10 moderate AP + 10 severe AP+ 15 controls; cell experiment	Clin + Cae (*in vitro*)	Level 1 (Strong)	Decreased	miR-148a-3p,miR-148b-3p	Alleviation	Li et al. (2024)
	Circ_0000284	AP (early/acute phase)	AR42J Cell	Only cell experiments	Cae (*in vitro*)	Level 3 (Basic)	Increased	miR-10a-5p/Wnt/β-catenin axis	Aggravatin	Huang et al. (2022)
	CircZFP644	AP (early/acute phase)	AR42J Cell	Only cell experiments	Cae (*in vitro*)	Level 3 (Basic)	Decreased	miR-106b/Pias3 Axis	Alleviation	Wang et al. (2021)
Apoptosis	CircZFP644	SAP	Mice	6 SAP +6 Control	Anim	Level 2 (Moderate)	Decreased	miR-21-3p	Aggravatin	Yang et al. (2020)
	CircDTNB	AP (early/acute phase)	AR42J Cell	Only cell experiments	Cae (*in vitro*)	Level 3 (Basic)	Decreased	microRNA-485-5p/MCL1 Axis	Alleviation	Tian et al. (2024)
Pyroptosis	CircHIPK3	AP (covering mild, and severe cases)	Human,AR42J Cell	72 patients +34 controls; cell experiment	Clin + Cae (*in vitro*)	Level 1 (Strong)	Increased	miR-193a-5p/GSDMD Axis	Aggravatin	Wang et al. (2020)
		AP	Mice, MPC-83 Cell	42 valid mice (7 groups × 6 mice); cell experiment	Anim + Cae (*in vitro*)	Level 2 (Moderate)	Increased	miR-193a-5p/NLRP3 Axis	Aggravatin	Feng et al. (2024)

### 6.1 Inflammation

The pathogenesis of pancreatitis mostly involves the abnormal activation of trypsinogen, which induces pancreatic cell damage, releases damage-associated molecular patterns (DAMPs), triggers the innate immune response, activates the inflammasome, and promotes the production of IL-1β (Yang et al., 2020; Kono and Rock, 2008). This additionally upregulates leukocyte adhesion molecules and facilitates neutrophil infiltration (Kubes and Mehal, 2012; Frossard et al., 1999). Additionally, pancreatitis can facilitate the interaction of microbiome-associated molecular patterns (MAMPs) with pathogen recognition receptors (PRRs) by modifying intestinal permeability, leading to the activation of NF-κB (Rakonczay et al., 2008) and fostering pancreatic inflammation (Gianotti et al., 1993; Rychter et al., 2009). These processes intensify the inflammatory response through pro-inflammatory cytokines (e.g., IL-6, IL-1β, TNF-α) (Mayer et al., 2000; Malmstrøm et al., 2012Malmstrøm et al., 2012) and chemokines (e.g., IL-8, MCP-1) (Malmstrøm et al., 2012; Regnér et al., 2008; Sakai et al., 2003; Watanabe et al., 2017).

#### 6.1.1 Circ_0073748

Significant upregulation of circ_0073748 was detected in the plasma and caerulein-stimulated pancreatic duct cells of AP patients. Circ_0073748 acts as a molecular sponge for miR-132-3p, and regulates TRAF3 expression. TRAF3 is a member of the TNF Receptor-Associated Factor (TRAF) family (Bishop, 2016) and plays a crucial role in immune signaling. Inhibiting of circ_0073748 expression diminishes its interaction with miR-132-3p, thereby enhancing the miRNA-mediated suppression of TRAF3 and ultimately reducing the activation of the NF-κB signaling pathway, which significantly mitigates pancreatic duct cell injury, inflammation, and oxidative stress (Ren et al., 2022). While these findings are mechanistically insightful, the evidence is primarily derived from *in vitro* Caerulein-induced models, and clinical validation in larger patient cohorts is warranted.

#### 6.1.2 Circ_UTRN

In pancreatic acinar cells, the overexpression of circ_UTRN acts as a molecular sponge for miR-320-3p, and alleviated the inhibition of PTK2 (Protein Tyrosine Kinase 2) by miR-320-3p, thereby enhancing the expression of PTK2 (Sun et al., 2022). Consequently, it reduces the secretion of inflammatory mediators (such as TNF-α, IL-1β, and IL-6) and suppresses the inflammatory response in pancreatic acinar cells. Thus, circ_UTRN also counteracts the apoptosis inhibition caused by caerulein and facilitates the removal of injured cells. This mechanismawaits validation in human tissues or *in vivo* models to confirm its translational potential.

#### 6.1.3 Circ_0000037

In the AP model, circ_0000037 functions as a sponge for miR-92a-3p, thereby relieving miR-92a-3p-mediated suppression of its target gene PIAS1, which ultimately upregulates PIAS1 expression. PIAS1 is a crucial anti-inflammatory protein whose overexpression mitigates inflammation and cell death. In pancreatitis, the expression of circ_0000037 is downregulated, whereas miR-92a-3p is upregulated, and PIAS1 is downregulated, thereby promoting apoptosis and inflammation in pancreatic cells. Thus, circ_0000037 alleviates AP progression by regulating the miR-92a-3p/PIAS1 axis (Sun et al., 2022). While, this axis is supported by functional assays in cellular models; however, clinical correlation and direct interaction evidence are lacking.

#### 6.1.4 circ_0029407

Li et al. discovered that circ_0029407 was markedly elevated in the serum of AP patients and in human pancreatic epithelial cells stimulated by hyaluronan. This finding provides stronger evidence by combining patient-derived data with *in vitro* validation. Inhibiting the expression of circ_0029407 alleviates cell damage induced by hyaluronan, thereby promoting cell proliferation, inhibiting apoptosis, and reducing the secretion of pro-inflammatory cytokines. Subsequent research has demonstrated that circ_0029407 acts as a competitive endogenous RNA (ceRNA) for miR-579-3p to modulate the TLR4/NF-κB signaling pathway,. thereby influencing the inflammatory response and the damage of pancreatic cells (Lu et al., 2024; Ma et al., 2023).

#### 6.1.5 circMAP3K5

Research indicates that the expression level of circMAP3K5 in patients with AP is significantly downregulated and inversely correlated with disease severity. This observed correlation enhances the clinical relevance of the findings. Mechanistic studies revealed that circMAP3K5 functions as a ceRNA, primarily by sponging miRNAs such as miR-148a-3p and miR-199a-5p. Through this mechanism, circMAP3K5 overexpression alleviates the repression of target genes, leading to a marked reduction in the production of inflammatory factors such as IL-1β, IL-6, and TNF-α (Li et al., 2024). Further *in vivo* functional studies are warranted to establish a causal relationship.

#### 6.1.6 circ_0000284

Circ_0000284 promotes the pathogenesis of AP by sponging miR-10a-5p, which leads to activation of the Wnt/β-catenin signaling pathway, thereby enhancing inflammation and inhibiting apoptosis. The Wnt/β-catenin pathway is a signaling cascade consisting of a collection of proteins that is crucial for embryonic development and the maintenance of adult tissue homeostasis (Liu et al., 2022). The canonical Wnt/β-catenin pathway regulates cell proliferation, survival, differentiation, and migration through the nuclear translocation of β-catenin and the activation of TCF/LEF transcription factors (Niehrs, 2012). Experiments demonstrated that circ_0000284 sponges miR-10a-5p, thereby activating the Wnt/β-catenin pathway, and exacerbating inflammation and apoptosis (Huang et al., 2022). Current evidence is based on experimental models; clinical association and pathway specificity require further investigation.

​​ In conclusion, circRNAs are of great significance in the occurrence and development of pancreatitis. They regulate AP pathogenesis by acting as competing endogenous RNAs (ceRNAs) to modulate inflammatory responses. Protective circRNAs (e.g., circ_0073748, circ_0000037, circ_0029407, circMAP3K5) suppress inflammation via miRNA sponging: circ_0073748 upregulates TRAF3 to inhibit NF-κB, circ_0000037 elevates PIAS1 to reduce apoptosis, circ_0029407 blocks TLR4/NF-κB via miR-579-3p, and circMAP3K5 sequesters miR-148a-3p/miR-199a-5p to curb cytokine release. Conversely, pro-inflammatory circRNAs (circ_UTRN, circ_0000284) exacerbate damage: circ_UTRN promotes PTK2 expression via miR-320-3p sponging, while circ_0000284 activates Wnt/β-catenin signaling by sponging miR-10a-5p. Notably, the level of supporting evidence varies considerably across these proposed axes. Findings from patient-derived samples (e.g., circ_0029407, circMAP3K5) carry greater translational weight, whereas mechanisms solely established in Caerulein-induced models (e.g., circ_0073748, circ_UTRN, circ_0000037) require further clinical correlation and validation in more physiologically relevant systems.

Despite these insights, the field faces several limitations: the predominant reliance on caerulein-induced cell/animal models limits clinical translatability; small patient cohorts and the use of unvalidated functional assays (e.g., lack of CRISPR (Clustered Regularly Interspaced Short Palindromic Repeats) validation for circ_0000037/PIAS1 interactions) compromise the robustness of the findings; and static analyses overlook dynamic ceRNA networks across disease stages. Future work should utilize patient-derived organoids, integrate multi-omics data, and develop targeted therapeutics (e.g., circRNA-specific ASOs) to bridge these gaps, thereby accelerating the translation of circRNA research into clinical applications for AP.

### 6.2 Apoptosis

Apoptosis regulates programmed cell death through intrinsic and extrinsic pathways involving key regulators such as Bcl-2 family proteins and caspases (Gon et al., 1996; Liu et al., 1996). Apoptosis is characterized by DNA fragmentation, vesiculation, and the release of phagocytic signals (Katsumori et al., 2011; Ravichandran, 2010), facilitating the clearance of the cell in a non-inflammatory way. Recent findings indicate that circRNAs modulate apoptotic pathways by interacting with apoptosis-related molecules, suggesting their significant involvement in pancreatitis pathogenesis. The core mechanisms of AP encompass aberrant trypsinogen activation (Aghdassi et al., 2018), dysregulated calcium signaling (Wen et al., 2015), and mitochondrial dysfunction (Biczo et al., 2018). Recent findings indicate that circRNAs may be involved in regulating the apoptosis of pancreatic acinar cells through regulating calcium homeostasis and mitochondrial activity.

#### 6.2.1 circZFP644

MiR-21-3p is elevated in severe acute pancreatitis (SAP) and correlates with the suppression of apoptosis and the enhancement of necrosis in pancreatic cells. CircZFP644 functions as a competitive endogenous RNA (ceRNA) by binding to miR-21-3p, thereby diminishing its expressionand mitigating its inhibitory effect on apoptosis, which ultimately reduces pancreatic damage and necrosis (Yang et al., 2020). In addition, Wang et al. discovered that the expression of circZFP644 was significantly downregulated in AP models, and the overexpression of circZFP644 suppressed the secretion of inflammatory factors (TNF-α, IL-1β, IL-6), while promoting apoptosis. Mechanism investigations indicate that circZFP644 modulates Pias3 expression via targeting miR-106b, and the inhibition of miR-106b can further diminish the inflammatory response and augment apoptosis. These findings indicate that circZFP644 in AP possesses anti-inflammatory properties and facilitates apoptosis, demonstrating a dual role (Wang et al., 2021). These conclusions are drawn primarily from cell line models (e.g., AR42J); human tissue validation and *in vivo* functional studies are necessary to confirm pathophysiological relevance.

#### 6.2.2 circDTNB

In AP models of the pancreatic acinar cell line AR42J, the downregulation of circDTNB was observed to exacerbate inflammation and apoptosis. Subsequent research indicated that circDTNB alleviated the suppressive effect of miR-485-5p on MCL1 (myeloid cell leukemia 1) by competitively interacting with miR-485-5p, thereby diminishing cytotoxicity and inflammatory response (Tian et al., 2024). MCL1 is a crucial anti-apoptotic protein of Bcl-2family, capable of inhibiting the pro-apoptosis proteins Bax and Bak to prevent the formation of the mitochondrial apoptosis-induced channel, hence ensuring cell survival. It is significant in B cells, T cells, and neurons, and plays a vital role in the development of hematopoietic stem cells (Thomas et al., 2010). These results indicate that circDTNB serves a protective role in AP through the miR-485-5p/MCL1 axis. This mechanism is supported by *in vitro* evidence but lacks correlation with human AP samples or *in vivo* validation.

CircRNAs intricately modulate pancreatic acinar cell apoptosis in AP through ceRNA networks targeting miRNAs linked to mitochondrial dysfunction and calcium signaling. CircZFP644 acts as a pro-apoptotic factor by sponging miR-21-3p to upregulate Pias3, enhancing caspase-dependent cell death and reducing necrosis (Yang et al., 2020; Wang et al., 2021), while circDTNB protects cells by sequestering miR-485-5p to stabilize MCL1, inhibiting Bax/Bak-mediated apoptosis (Tian et al., 2024; Thomas et al., 2010). The evidence supporting these apoptotic regulatory networks remains predominantly experimental and preclinical. The current findings, largely derived from caerulein-induced AR42J cell models and not validated in primary human acinar cells or tissues, limit their clinical generalizability.

Despite their therapeutic potential, current evidence is limited by reliance on caerulein-induced AR42J cell models and lacks direct human tissue validation, raising concerns about translational relevance. Mechanistic gaps persist, including incomplete downstream apoptosis pathway characterization (e.g., caspase activity) and unverified specificity of circRNA-miRNA interactions. Inconsistent expression patterns further challenge reproducibility. Future studies should employ human-derived organoids, multi-omics integration, and standardized apoptosis assays to enhance rigor, while therapeutically targeting circRNAs with chemically modified mimics/inhibitors may bridge bench-to-bedside translation.

### 6.3 Pyroptosis

Pyroptosis is a form of programmed cell death initiated by the activation of inflammasomes, such as NLRP3 (NACHT, LRR and PYD domains-containing protein 3), which activates caspase-1 and cleaves gasdermin D (GSDMD). This process intensifies tissue damage by producing inflammatory mediators such as IL-1β (Boucher et al., 2018). In AP, the NLRP3/caspase-1/GSDMD axis induces pyroptotic death and exacerbates systemic inflammation (Gao et al., 2021), whereas the inhibition of caspase-1 significantly alleviates inflammation in the AP model (Zhang et al., 2014). Recent studies have demonstrated that circRNAs not only regulate thepancreatic cell death but also influence pyroptosis by modulating inflammasome activity and key pyroptotic factors, such as caspase-1 and GSDMD.

#### 6.3.1 circHIPK3

Research indicates that circHIPK3 facilitates pyroptosis and inflammation in pancreatic cells by disrupting the function of miR-193a-5p. Specifically, circHIPK3 functions as a molecular sponge for miR-193a-5p, resulting in reduced miRNA levels and subsequent disinhibition of GSDMD. GSDMD is a key executor of protein-mediated pyroptosis (Balasubramanian et al., 2024). Upon GSDMD activation, cells undergo pyroptosis, releasing numerous inflammatory factors (such as IL-1β, IL-6, IL-8, and TNF-α) and further amplifying the inflammatory response (Wang et al., 2020). Moreover, the molecular sponging activity of circHIPK3 alleviates the inhibitory effect of miR-193a-5p on the NLRP3 inflammasome, leading to its activation, which then promotes the activation of caspase-1 and GSDMD, thereby enhancing pyroptosis and inflammation. In conclusion, targeted inhibition of circHIPK3 can significantly diminish the inflammatory response in AP (Feng et al., 2024). The evidence for this axis is derived from mechanistic studies in cellular models; its operation in human disease contexts and potential as a biomarker or therapeutic target requires further investigation in clinical cohorts.

## 7 CircRNAs across the spectrum of pancreatic diseases

While the roles of circRNAs in AP are increasingly being elucidated, their functions in chronic pancreatitis (CP) and pancreatic cancer (PC) present a more complex and evolving picture. This comparative analysis aims to situate the findings in AP within the broader context of pancreatic pathologies, highlighting both conserved and divergent mechanisms.

### 7.1 AP vs. CP: Bridging the gap via fibrosis

A striking gap exists in the literature regarding circRNAs’ expression and function specifically in CP. Unlike AP, which is characterized by sudden inflammation and injury, CP involves persistent, fibrotic destruction of the pancreas. We propose that fibrosis pathways involving TGF-β and pancreatic stellate cell (PSC) activation are highly pertinent to CP pathogenesis. Given the established roles of circRNAs in regulating TGF-β signaling and fibrosis in other organ systems (Dai et al., 2021; Jiang and Ning, 2019), it is highly plausible that they contribute to CP pathogenesis by modulating PSC activation and extracellular matrix deposition. The absence of studies directly linking circRNAs to CP underscores a significant opportunity for future research, potentially identifying circRNAs that differentiate acute flare-ups from chronic background inflammation or that predict progression to fibrosis.

### 7.2 AP vs. PC: The inflammatory-oncogenic nexus

The circRNAs’ landscape in PC is extensively studied, often revealing roles in proliferation, invasion, and metastasis—processes less relevant to AP. However, intriguing parallels exist, particularly through shared inflammatory-oncogenic pathways. A key link involves the NLRP3 inflammasome and the glycolysis/PKM2 axis. For example, circHIPK3 is upregulated in both AP and PC. In AP, it promotes NLRP3 inflammasome-mediated pyroptosis and inflammation (Gao et al., 2021), whereas in PC, circERC1 inhibits pyroptosis—a pro-inflammatory cell death process—by disrupting the hnRNPA1-PKM2-NLRP3 axis, thereby promoting tumor cell survival and drug resistance (Gu et al., 2022). This suggests that circRNAs can exert context-dependent functions, promoting inflammation in AP and supporting survival in PC. This nexus between inflammation, metabolic reprogramming (glycolysis/PKM2), and cancer risk provides a fertile ground for discovering circRNAs that bridge acute injury and malignant transformation.

### 7.3 Conclusion and future perspectives

In summary, circRNAs in AP primarily act as mediators of rapid inflammatory response and cell death, while in PC, they are often co-opted to drive sustained growth and survival. The role in CP remains largely hypothetical but likely involves regulating fibrotic and chronic inflammatory processes. Future studies should prioritize profiling circRNAs’ expression in CP patient samples. Verifiable predictions include: (1) identifying a CP-specific circRNA signature that differentiates it from AP and PC; (2) determining whether circRNAs associated with AP resolution (e.g., those with anti-inflammatory effects) are dysregulated in CP could reveal mechanisms underlying disease chronicity. Promising candidate circRNAs for CP research include those known to regulate TGF-β signaling (e.g., circTGFBR2, circSMAD2) or macrophage polarization (e.g., circRNA_0057344) in other fibrotic diseases. Similarly, exploring if certain “pro-inflammatory” circRNAs in AP share identity with “oncogenic” circRNAs in PC could provide insights into the molecular link between inflammation and cancer, potentially identifying shared therapeutic targets for the entire pancreatic disease spectrum.

## 8 Summary and outlook

As an important regulator of AP, circRNAs have shown great potential as a biomarker and therapeutic target in multiple studies. For example, their inherent biophysical properties—including high abundance in body fluids, exceptional stability due to the covalently closed circular structure, and often tissue-specific expression patterns—make them particularly suitable as non-invasive diagnostic tools (Liu C. et al., 2020; Wang et al., 2020). In addition, the expression changes of these circRNAs can reflect disease progression or treatment response, offering a promising approach for monitoring therapeutic efficacy. Beyond diagnostic applications, circRNAs have also emerged as potential therapeutic targets by modulating signaling pathways associated with inflammatory responses, pyroptosis, and apoptosis (Lu et al., 2024; Wang et al., 2021). Although many findings of circRNAs’ involvement in AP regulation have been reported, most of them are preliminary observations. For example, many studies in the literature have focused on the circRNA/miRNA/mRNA axis, but the regulatory mechanisms upstream of circRNAs are lacking. Only one article reported the relationship between m6A modification of circRNAs in a mouse SAP model (Wu et al., 2021); however, the role of m6A modification in other forms of AP and its consistency between human patients and mouse models remain unexplored. Additionally, the same circRNAs can exert opposing functions when binding to different downstream factors to affect AP, but there is a lack of research on the cooperation and competition between downstream factors (Yang et al., 2020; Wang et al., 2021). Compared to fields like cancer (Hang et al., 2018; Li P. et al., 2015) or cardiovascular diseases (Ruberto and Foo, 2025)where circRNAs biogenesis regulation, specific functional mechanisms (Chen, 2020), and extensive clinical validation studies are more advanced, research on circRNAs in AP is still in its relatively early stages. Future AP research should prioritize elucidating these upstream regulatory mechanisms and resolving the context-dependent functions of individual circRNAs.

To bridge these gaps, future studies should prioritize the following: (1) Utilizing more physiologically relevant human model systems, such as patient-derived organoids or primary acinar/stellate cell cultures, to reduce reliance on caerulein-based rodent models and enhance clinical translatability. (2) Applying multi-omics approaches (epitranscriptomic, proteomic, and single-cell transcriptomic) to map upstream regulators (e.g., RNA-binding proteins like QKI or METTL3/14) that control circRNA expression and modification in AP. (3) Conducting large-scale, multi-center clinical validation studies to correlate specific circRNAs (e.g., circHIPK3, circ_0073748) with standardized clinical outcomes across diverse AP cohorts. (4) Developing circRNA-targeting therapeutics, such as CRISPR-based knockdown or nanoparticle-delivered circRNA mimics/inhibitors, and testing their efficacy in well-characterized preclinical models of AP.

At present, although there are many studies on circRNAs and AP, there are few studies on circRNAs and chronic pancreatitis (CP). CP is characterized by gland fibrosis, which is markedly different from the glandular inflammatory tissue of AP. There is still a lack of in-depth research on circRNAs and CP, and more studies are urgently needed to clarify the mechanism of action of circRNAs in chronic lesions and open up new horizons for the treatment of pancreatic disease. Future studies on circRNAs in CP could focus on their roles in pancreatic stellate cell activation (Liu et al., 2024), extracellular matrix remodeling, and persistent inflammation, drawing parallels and distinctions from circRNAs functions established in fibrosis of other organs like liver or kidney.

Further clinical validation of these findings is essential, particularly through confirmatory studies in large cohorts, in-depth mechanistic dissection of circRNA functions, and the development of circRNA-targeted therapeutics, which will be key focuses of future research (Chen R. et al., 2023). Moreover, integrating circRNAs with other molecular markers or therapeutic targets could enhance diagnostic accuracy and treatment efficacy. Leveraging advanced technologies like single-cell sequencing to map cell type-specific circRNA expression patterns during disease progression, and developing novel delivery systems for pancreatic-targeted circRNA therapeutics, represent critical future directions (Shrinivas et al., 2019). The application of single-cell sequencing and spatial transcriptomics could further resolve cell type-specific circRNA networks and interaction maps during AP progression and the transition to CP. Furthermore, systematically investigating the dynamic changes in circRNA regulatory networks across the AP-CP continuum could reveal key drivers of disease progression.

Overall, circRNAs provide a new perspective for the diagnosis and treatment of AP. With the advancement of molecular biology technology, circRNAs is expected to become an important tool in precision medicine, promoting personalized diagnosis and treatment of patients with AP, enabling earlier disease detection and more effective interventions.
